# Interactions Between Transfemoral Amputees and a Powered Knee Prosthesis During Load Carriage

**DOI:** 10.1038/s41598-017-14834-7

**Published:** 2017-11-03

**Authors:** Andrea Brandt, Yue Wen, Ming Liu, Jonathan Stallings, He Helen Huang

**Affiliations:** 1 University of North Carolina, Joint Department of Biomedical Engineering, Chapel Hill, Raleigh, 27514 USA; 2 North Carolina State University, Joint Department of Biomedical Engineering, Chapel Hill, Raleigh, 27695 USA; 3 North Carolina State University, Department of Statistics, Raleigh, 27695 USA

## Abstract

Machines and humans become mechanically coupled when lower limb amputees walk with powered prostheses, but these two control systems differ in adaptability. We know little about how they interact when faced with real-world physical demands (e.g. carrying loads). Here, we investigated how each system (i.e. amputee and powered prosthesis) responds to changes in the prosthesis mechanics and gravitational load. Five transfemoral amputees walked with and without load (i.e. weighted backpack) and a powered knee prosthesis with two pre-programmed controller settings (i.e. for load and no load). We recorded subjects’ kinematics, kinetics, and perceived exertion. Compared to the no load setting, the load setting reduced subjects’ perceived exertion and intact-limb stance time when they carried load. When subjects did not carry load, their perceived exertion and gait performance did not significantly change with controller settings. Our results suggest transfemoral amputees could benefit from load-adaptive powered knee controllers, and controller adjustments affect amputees more when they walk with (versus without) load. Further understanding of the interaction between powered prostheses, amputee users, and various environments may allow researchers to expand the utility of prostheses beyond simple environments (e.g. firm level ground without load) that represent only a subset of real-world environments.

## Introduction

Daily use of modern, wearable, assistive devices (e.g. powered prostheses, powered exoskeletons) requires the mechanical coupling of the machine and human user. The control systems of machines and humans greatly differ in their ability to adapt to various physical demands typically experienced in daily life (e.g. avoiding obstacles, adjusting to changes in ground compliance, adjusting to different loads). The central nervous system allows a person to rapidly adjust to changes in physical environments and learn new tasks through advanced sensory feedback processing. On the other hand, people typically program machines to execute well-studied tasks, with limited adaptability to unexpected variations of those tasks. When a human and machine are mechanically coupled, a complex system emerges. Each part, with varying levels of adaptability, must take into consideration the dynamics of their counterpart when executing a task, rather than acting independently. Wearable powered devices must coordinate with the user to simultaneously and safely adjust to changes in the environment, while manipulating only a limited number of joints practical for the coupled system (i.e. number of biological joints requiring assistance or substitution). In order to advance the usefulness of powered assistive devices in the real world, researchers should first understand how the devices and users interact in various environments.

One example of a coupled human and machine executing a task is the coupling of a transfemoral amputee with a powered knee prosthesis during steady-state walking. Steady-state walking is a well-studied and cyclic movement, so preprogrammed powered prostheses can mimic steady-state walking in simple environments (e.g. firm level ground with no other environmental interactions). Current powered knee prostheses emulate biological lower limb muscle function during walking by modulating the prosthetic knee joint impedance during predefined gait phases via a finite-state impedance controller^1–3^. The impedance control parameters must be personalized to account for functional variations across amputee users (e.g. body weight, hip range of motion). In their current practice, clinicians manually fine-tune the impedance control parameters for each amputee user as they walk on level ground in a clinic^4^, and the fine-tuned parameters remain fixed for daily use. Consequently, these pre-programed prosthesis controllers have limited ability to adapt to changes in environmental and user demands in the real world, in turn limiting lower limb amputees’ performance level of daily activities with their prosthesis.

Previously, researchers have studied the interaction between powered prostheses and amputee users by experimentally manipulating prosthesis control parameters and evaluating amputees’ gait performance as they walked at a steady state in a simple environment. Some researchers used this method to explore ways to make the tuning procedure more efficient and objective^5–9^ and to identify more functionally beneficial control parameters and transition timings (e.g. reduced metabolic cost, reduced disturbance to user’s balance)^10–14^. With appropriate fundamental control parameters, powered prostheses can improve amputee user satisfaction, performance, and metabolic cost (though these measures are not necessarily correlated)^11^. These studies demonstrate the need to understand amputee-prosthesis interactions in order to develop prosthesis controllers that effectively adapt to the needs of individual amputee users. However, prior studies of amputee-prosthesis interactions have not included real-world physical demands users might experience outside of the clinic. Varol *et al*. (2010) tested the robustness of their user intent recognition controller that switches activity modes (e.g. standing to walking) of a powered prosthesis by observing its performance as users carried load, but they did not examine user performance within activity modes (e.g. walking)^15^. From the user’s perspective, his/her quality of life is strongly associated with his/her prosthesis performance during daily activities (e.g. walking while carrying load, walking on slippery surfaces)^16^. Lower limb amputees experience social and psychological benefits (e.g. confidence in one’s own ability to accomplish an activity) with greater independence and social engagement^17^. Yet, no studies to our knowledge have investigated the performance of powered prostheses and their users subjected to real-world environmental demands.

Walking with load is a particularly challenging and common real-world physical demand for lower limb amputees^16^. Walking with just 10 pounds of groceries, for example, may lead transfemoral amputees to sacrifice their prosthesis for the security of a wheelchair for this task^18^. Beyond carrying everyday items such as groceries, people frequently carry loads for occupational, recreational, and military purposes. Able-bodied people typically incur local physiological penalties when carrying load (e.g. increased muscle force demands^19^) that contribute to higher overall metabolic costs^20^. Goslin and Rorke (1986) found that perceived exertion positively correlated with central responses such as heart rate (r = 0.46) and oxygen consumption (r = 0.71) while able-bodied people walked with an additional load of 20% of their body mass^21^. Greater perceived exertion also moderately correlated with older adults’ lesser confidence in walking (r = −0.33) and greater fear of falling during daily activities (r = 0.26), which are important considerations for lower limb amputees^22^. On a local to global level, able-bodied people modulate their biological knee joint mechanics when carrying additional load in order to maintain their knee and center of mass excursions^23^. Lower limb amputees walking with passive prostheses, however, are only capable of making adjustments via their biological joints, as the prosthesis joint mechanics remain fixed. As a result, transtibial amputees walking with energetically-passive prostheses carrying load increase their reliance on their intact limb and their double support time (i.e. amount of time with both limbs on the ground), a common gait-stabilizing strategy^24^. Both transtibial amputees^25^ and transfemoral amputees^26^ demonstrate significantly higher metabolic costs compared to able-bodied people walking with identical loads. By modulating the joint mechanics, powered lower limb prostheses can reduce amputees’ metabolic cost and normalize gait during walking^27^, and potentially during load carriage as well^28^. The open question is whether adaptive control of powered prostheses in response to different external loads is beneficial for lower limb amputees.

Motivated by this research question, we investigated the influence of different powered knee mechanics (i.e. impedance control parameters fine-tuned for walking with load and no load) in transfemoral amputees walking with and without load. To evaluate the influence of load and powered knee mechanics, we analyzed human-prosthesis interaction effects at a local level (i.e. joint kinematics and kinetics, temporal measures) and a global level (i.e. perceived exertion and center-of-mass velocity). Center-of-mass velocity is a global measurement that incorporates inter-limb changes and is known to be asymmetric during mid-stance for amputees due to prosthesis deficiencies^29^. We anticipated that local changes such as more or less compensation from intact joints with each testing condition would also induce global changes due to the fixed powered knee mechanics. More specifically, we hypothesized that 1) when walking with mismatched testing conditions (i.e. walking without load and control parameters for load, walking with load and control parameters for no load), transfemoral amputees would perceive greater effort, rely more on their intact limb (i.e. increased intact-limb stance time, increased intact-limb joint work), increase double support time, and decrease intact-limb center-of-mass velocity, and that 2) these adverse effects would be mitigated with the use of prosthesis control parameters fine-tuned for the appropriate load condition. Our effort to investigate interactions between the prosthesis, human user, and environment may lead to a deeper understanding of prosthesis user behavior in the real world and whether load-specific prosthetic knee mechanics may be useful for daily use. Further, the results from this study may lay a foundation for more advanced powered prosthesis controllers that can effectively adapt to various environmental demands.

## Results

### Powered knee impedance control parameters

From the standard set of prosthetic knee impedance control parameters that were provided at the beginning of each tuning session, the expert adjusted the impedance control parameters for every subject to optimize their observable gait performance. The tuning procedure was based on the procedure used in clinics for powered prosthesis tuning (see Methods). Between the two tuned impedance parameter sets (i.e. tuned for no load and load walking), the expert consistently arrived at different stiffnesses and/or equilibrium positions for the stance extension phase (Supplementary Table S1). The magnitude of each parameter adjustment varied across subjects. Compared to the *no load* impedance parameter set, the *load* impedance parameter set included 9 ± 0.1% greater stiffness across subjects and 29 ± 0.2% greater equilibrium position.

### Kinematics

**Figure 1:**
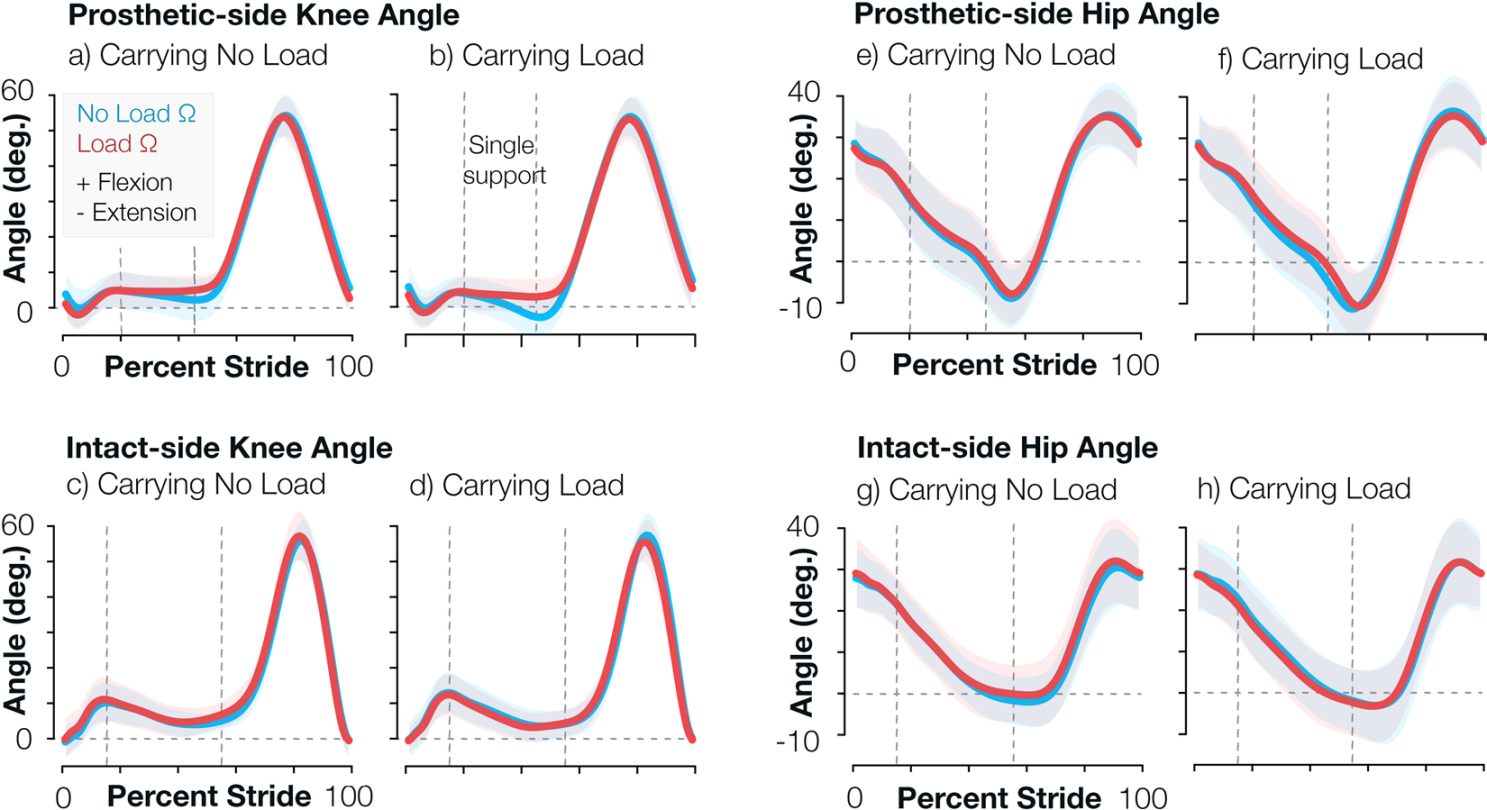
Prosthetic knee angle changed primarily during load carriage, and all other kinematics remained relatively unchanged. Knee and hip angle trajectories plotted for both limbs and all 4 testing conditions. Each plot compares the performance of the *no load* impedance parameters (blue) and *load* impedance parameters (red) for different joints and load conditions. Shaded regions illustrate one standard deviation across subjects. Vertical dotted lines indicate contralateral toe off and heel strike timing, so the region between the two lines is the ipsilateral limb’s single support time. Positive angles represent joint flexion, and negative angles represent joint extension.

By design, the powered knee prosthesis performance was highly dependent on the predetermined impedance parameters (1) as well as the amputee subjects’ interaction (e.g. loading) with the device. The resulting prosthetic knee angle was a product of both the powered prosthesis and amputee subject’s efforts. All changes in the powered knee angle occurred during mid- to late-stance when the knee extended behind the amputee subjects’ torso (Fig. 1a,b). The impedance parameter set and load determined the magnitude of peak powered knee extension during stance, which was supported by a significant interaction effect of load and impedance (F(1,50) = 6.41, p = 0.015).

Specifically, when subjects walked without load and matched *no load* impedance parameters, the prosthetic knee resembled a normative knee angle (i.e. peak extension angle of 0 during late stance) with a mean peak extension angle of 2 ± 6 degrees across subjects. When subjects walked without load and *load* impedance parameters (i.e. mismatched condition), the knee angle was slightly more flexed than the matched condition with a mean peak extension angle of 4 ± 5 degrees (p = 0.142). When subjects walked with load and matched *load* impedance parameters, the prosthetic knee again resembled a normative knee angle with a mean peak extension angle of 2 ± 5 degrees. However, when subjects walked with load and mismatched *no load* impedance parameters, the prosthetic knee joint significantly hyper-extended compared to the matched condition with a mean peak extension angle of −3 ± 6 degrees (p < 0.001). The presence of load only significantly affected the prosthetic knee angle with *no load* impedance parameters (*no load* impedance p < 0.001, *load* impedance p = 0.250).

Amputee subjects’ intact joints did not exhibit significant kinematic changes between testing conditions, except for the prosthetic-side hip, which is an intact joint (Fig. 1, Supplementary Fig. S1). The peak extension angle of the prosthetic hip only exhibited a significant load main effect (F(1,50) = 10.49, p = 0.002). When amputee subjects walked with *load* impedance parameters, the presence of load increased the peak hip extension angle from −5 ± 7 to −8 ± 8 degrees (p = 0.012).

### Perceived exertion

**Figure 2:**
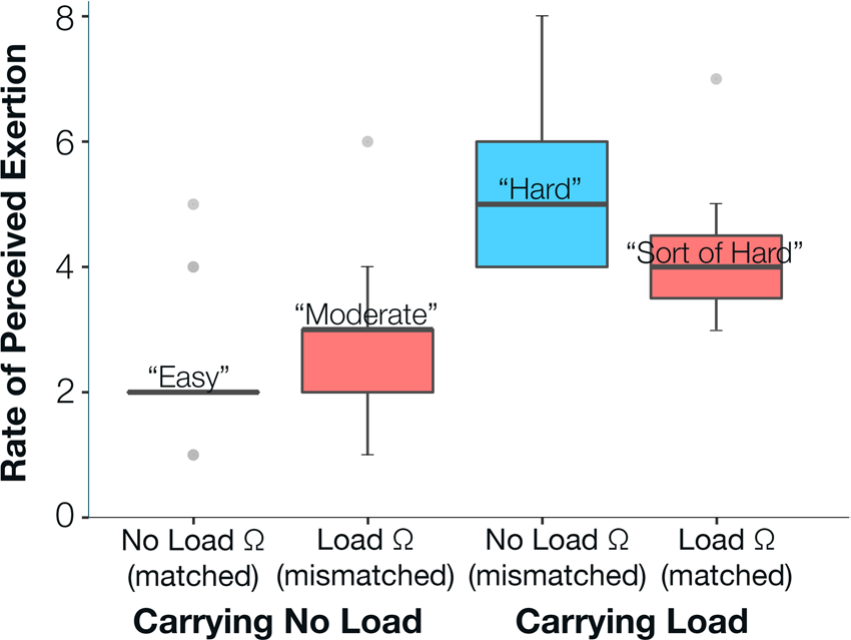
Both load and prosthetic knee mechanics significantly affected subjects’ rate of perceived exertion. Rate of perceived exertion distribution summary of all subjects shown for all four testing conditions. Red indicates *load* impedance parameters, and blue indicates *no load* impedance parameters. Matched conditions were conditions in which amputee subjects walked with powered knee impedance parameters tuned for the load they were carrying. Mismatched conditions were conditions in which amputee subjects walked with powered knee impedance parameters not tuned for the load they were carrying. Gray dots beyond the whiskers are data points greater than 1.5 times the interquartile range (i.e. colored regions).

All amputee subjects perceived on average 100% greater effort when walking with load compared to no load (Fig. 2). The impedance parameters also influenced amputee subjects’ perceived effort, evident by the significant interaction effect with load and impedance (F(1,50) = 8.62, p = 0.005). Specifically, when amputee subjects walked with load, they perceived significantly less exertion when walking with matched impedance parameters, with an average score of 4 ± 1 with *load* impedance and 5 ± 1 with *no load* impedance (p = 0.004). When they walked without load, amputee subjects perceived similar amounts of exertion, 2 ± 1, when they walked with both impedance parameter sets (p = 0.926). Using the same impedance parameters, amputee subjects perceived significantly greater exertion in the presence of load (*no load* impedance p < 0.001; *load* impedance p < 0.001).

### Temporal gait parameters

All amputee subjects maintained a relatively constant stride time between testing conditions (Table 1). Within each stride, subjects increased both their prosthetic-limb stance time (*no load* impedance p = 0.003; *load* impedance p < 0.001) and intact-limb stance time (*no load* impedance p < 0.001; *load* impedance p = 0.036). Swing time thus decreased with load on both the prosthetic limb (*no load* impedance p = 0.003; *load* impedance p < 0.001) and intact limb (*no load* impedance p < 0.001; *load* impedance p = 0.010). Amputees typically walk with a longer intact-limb stance time and shorter intact-side swing time (i.e. prosthetic-limb single support time). When subjects carried load, intact-limb stance time decreased (p < 0.001) and swing time increased (p < 0.001) with matched *load* impedance parameters. Intact-limb stance and swing time did not significantly differ when subjects did not carry load, and prosthetic-limb stance time did not significantly differ within either load condition (p > 0.100).

**Table 1:** Both load and prosthesis impedance parameters largely affected subjects’ temporal gait parameters. The first 4 columns summarize the average of each temporal parameter for each testing condition across subjects (mean±s.d.). Values are normalized to stride time of the corresponding limb. The last 3 columns summarize the factor effects of carrying load and altering the prosthesis impedance control parameters (i.e. p-value from ANOVA, 50 denominator degrees of freedom). Statistically significant changes are in bold font. Conditions without the same superscript letter (a–c) are significantly different at the 0.05 level with Tukey’s multiple comparisons adjustment.

	No Load		Load		load Main Effect	Impedance Main Effect	Interaction Effect
	Impedance for no load	Impedance for load	Impedance for no load	Impedance for Load			
***Stride Time (s)***							
*(prosthetic)*	1.7 ±0.1	1.7 ± 0.1	1.7 ± 0.1	1.7 ± 0.1	0.177	0.509	0.141
*(intact)*	1.7 ± 0.1	1.7 ± 0.1	1.7 ± 0.1	1.7 ± 0.1	0.152	0.522	0.137
***Stance Time (%)***							
*(prosthetic)*	59 ±2^b^	59 ±2^b^	60 ±3^a^	61 ±2^a^	**<0.001**	0.150	0.132
*(intact)*	76 ± 2^bc^	76 ± 2^c^	77 ± 2^a^	76 ± 2^b^	**<0.001**	<**0.001**	**0.010**
***Swing Time (%)***							
*(prosthetic)*	41 ± 2^a^	41 ± 2^a^	40 ± 3^b^	39 ± 2^b^	<**0.001**	0.150	0.132
*(intact)*	24 ± 2^ab^	24 ± 2^a^	23 ± 2^c^	24 ± 2^b^	<**0.001**	<**0.001**	0.054
***Total Double***							
***Support Time (%)***	35 ± 3^b^	35 ± 4^b^	37 ± 4^a^	37 ± 3^a^	<**0.001**	**0.035**	0.938
***Initial Double***							
***Support Time (%)***							
*(prosthetic)*	20 ± 3^b^	20 ± 2^b^	20 ± 2^b^	21 ± 2^a^	<**0.001**	<**0.001**	**0.028**
*(intact)*	15 ± 2^b^	14 ± 2^c^	17 ± 2^a^	15 ± 2^b^	**<0.001**	<**0.001**	0.051
***Terminal Double***							
***Support Time (%)***							
*(prosthetic)*	15 ± 2^b^	14 ± 2^c^	17 ± 2^a^	15 ± 2^b^	<**0.001**	**<0.001**	0.051
*(intact)*	20 ± 3^b^	20 ± 2^b^	20 ± 2^b^	21 ± 2^a^	**<0.001**	**<0.001**	**0.028**
***Step Time (%)***							
*(prosthetic)*	56 ± 1^b^	55 ± 1^c^	57 ± 2^a^	55 ± 1^c^	0.057	**<0.001**	**<0.001**
*(intact)*	44 ± 1^b^	45 ± 1^a^	43 ± 2^c^	45 ± 1^a^	0.127	<**0.001**	<**0.001**
***Step Time***							
***Asymmetry***							
***Index (%)***	24 ± 5^b^	21 ± 6^c^	29 ± 7^a^	19 ± 6^c^	0.089	<**0.001**	<**0.001**

Amputee subjects’ total double support time significantly increased with load (*no load* impedance p < 0.001; *load* impedance p < 0.001), which is a common behavior for both people with and without amputation walking with load^20,24,30,31^. Our amputee subjects walked with a longer intact-limb terminal double support time (i.e. prosthetic-limb initial double support time) than prosthetic-side terminal double support time (i.e. intact-limb initial double support time) (Table 1). When amputee subjects carried load, total double support time remained relatively constant, but the matched *load* impedance parameters (compared to mismatched) increased intact-limb terminal double support time (p < 0.001) and decreased prosthetic-limb terminal double support time (p < 0.001). When they did not carry load, the matched *no load* impedance parameters increased prosthetic-limb terminal double support time (p = 0.014).

All amputee subjects exhibited greater prosthetic-side step time (i.e. terminal double support time and swing time combined) compared to intact-limb step time. For both load conditions, subjects’ prosthetic-side step time significantly decreased and intact-side step time significantly increased when subjects walked with *load* impedance parameters compared to *no load* impedance parameters (all pairwise comparisons p < 0.001). When walking with the *no load* impedance parameters only, intact-side step time significantly decreased (p < 0.001) and prosthetic-side step time significantly increased (p < 0.001) with the presence of load. Thus, the *load* impedance parameters allowed more symmetric step times (2) for both load conditions (both load conditions p < 0.001), but the difference in subjects’ step time when waking with matched and mismatched impedance parameters was much greater when subjects carried load versus no load.

### Kinetics

Intact-side joint kinetics were relatively unaffected by testing condition (Supplementary Fig. S1 and S2), but we observed small changes in joint work with testing conditions (Table 2). Joint work summarized the amputee subjects’ change in kinetic behavior across gait phases. Here we report specific pairwise comparisons with p < 0.10. When amputee subjects walked with *load* impedance parameters, the presence of load significantly decreased subjects’ positive ankle work (p = 0.028), moderately increased (in magnitude) subjects’ negative intact knee work (p = 0.053), and significantly increased subjects’ negative intact-side hip work (p = 0.046). Similarly, when they walked with *no load* impedance parameters, the presence of load significantly increased subjects’ negative intact knee work (p = 0.019) and negative intact-side hip work (p = 0.002). When subjects carried load, the matched *no load* impedance parameters (compared to mismatched) significantly decreased positive prosthetic-side hip work (p = 0.049).

**Table 2:** Load and prosthesis impedance parameters moderately affected subjects’ joint work. The first 4 columns summarize joint work normalized to body mass (W/kg) and averaged across subjects for each testing condition (mean ± s.d.). The last 3 columns summarize the factor effects of carrying load and altering the prosthesis impedance control parameters (i.e. p-value from ANOVA, 50 denominator degrees of freedom). Statistically significant changes are in bold font. Conditions without the same superscript letter (a–c) are significantly different at the 0.05 level with Tukey’s multiple comparisons adjustment.

	No Load		Load		Load Main Effect	Impedance Main Effect	Interaction Effect
	Impedance for no load	Impedance for load	Impedance for no load	Impedance for load			
***Intact Ankle***							
*(positive)*	0.25 ± 0.07^a^	0.23 ± 0.08^a^	0.24 ± 0.08^ab^	0.22 ± 0.09^b^	**0.015**	0.198	0.119
*(negative)*	−0.17 ± 0.05^ab^	−0.19 ± 0.03^b^	−0.16 ± 0.04^a^	−0.18 ± 0.04^ab^	0.123	**0.030**	0.922
***Intact Knee***							
*(positive)*	0.10 ± 0.07^a^	0.09 ± 0.06^a^	0.10 ± 0.06^a^	0.09 ± 0.06^a^	0.150	**0.041**	0.748
*(negative)*	−0.05 ± 0.02^a^	−0.06 ± 0.02^ab^	−0.07 ± 0.02^b^	−0.07 ± 0.02^b^	<**0.001**	0.294	0.772
***Intact-side Hip***							
*(positive)*	0.15 ± 0.03^a^	0.16 ± 0.03^a^	0.17 ± 0.05^a^	0.15 ± 0.04^a^	0.721	0.387	0.105
*(negative)*	−0.02 ± 0.01^ab^	−0.02 ± 0.01^a^	−0.03 ± 0.02^c^	−0.03 ± 0.02^bc^	<**0.001**	0.148	0.428
***Prosthetic-side Hip***							
*(positive)*	0.14 ± 0.08^ab^	0.12 ± 0.07^ab^	0.14 ± 0.09^a^	0.12 ± 0.07^b^	0.612	**0.008**	0.349
*(negative)*	−0.06 ± 0.02^a^	−0.06 ± 0.01^a^	−0.07 ± 0.03^a^	−0.07 ± 0.02^a^	**0.011**	0.520	0.921

### Center-of-mass velocity

**Figure 3:**
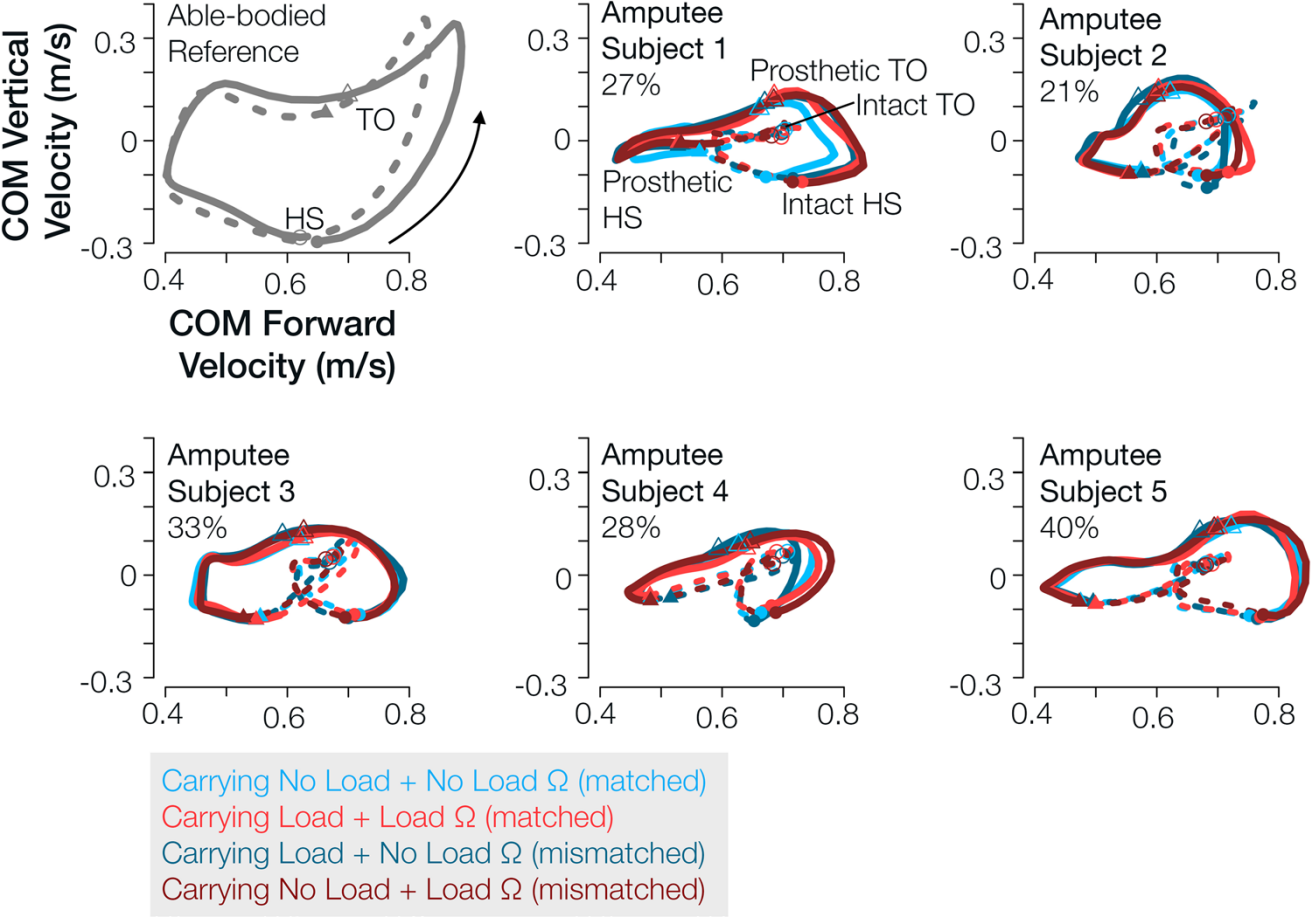
Center-of-mass forward and vertical velocity remained relatively unchanged between testing condition, but varied across subjects. Center-of-mass vertical velocity plotted against center-of-mass forward velocity for each amputee subject and testing condition. An able-bodied person’s center-of-mass hodograph included in top left (gray) for reference. Forward velocity asymmetry (2) during mid-stance is included as a percent in the top left of each plot. Each limb’s stance phase follows a counter-clockwise loop from heel strike to toe off. Filled point characters indicate heel strike and open point characters indicate toe off. Circles correspond to intact limb gait events, and triangles correspond to prosthetic limb gait events. Solid lines indicate intact-limb initial double support phase and single support phase, and dotted lines indicate prosthetic-limb initial double support phase and single support phase. Red indicates *load* impedance parameters, and blue indicates *no load* impedance parameters. Lighter colors correspond to the testing conditions in which amputee subjects did not carry load, and darker colors correspond to the testing conditions in which they carried load.

Amputee subjects’ forward and vertical center-of-mass velocity remained relatively unchanged between testing conditions (Fig. 3). Most notably, amputee subjects’ move their center of mass forward much faster during prosthetic-limb mid-stance compared intact-limb mid-stance, with varying degrees of asymmetry between subjects (Fig. 3). We calculated center-of-mass speed during mid-stance of each limb when vertical velocity was zero in order to capture the sagittal-plane effects related to prosthesis deficiencies^29^. Prosthetic-side forward velocity during mid-stance exhibited a significant interaction effect only (F (1,50) = 6.72, p = 0.013). Intact-side forward velocity during mid-stance did not exhibit any significant effects (p > 0.100). The comparison of the two conditions in which subjects walked with *no load* impedance parameters with and without load approached significance (p = 0.055), and all other comparisons were statistically insignificant (p > 0.200).

## Discussion

**Figure 4:**
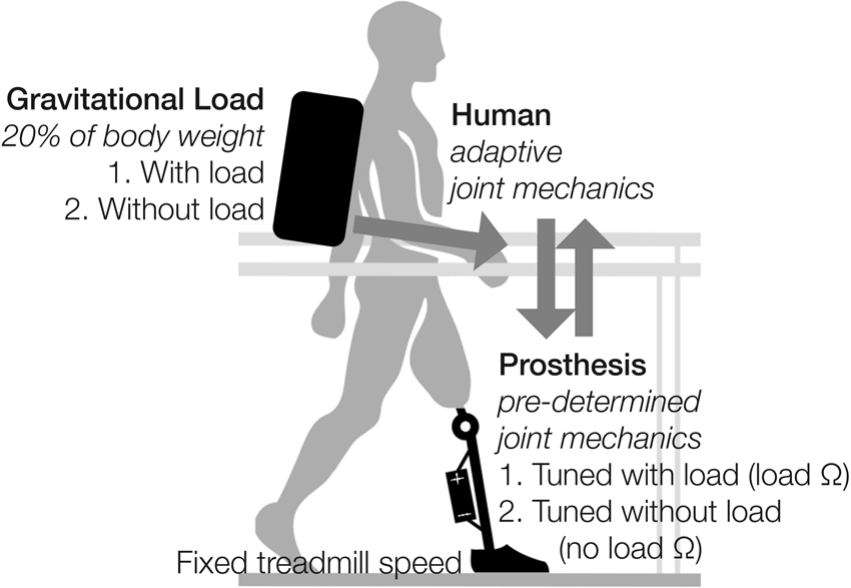
Experimental design concept. Amputee subjects walked at a fixed treadmill speed (0.6 m/s) with and without load and powered knee prosthesis joint mechanics tuned for each load, resulting in 4 load-mechanics testing combinations. We investigated the effects of gravitational load (i.e. weighted backpack, 20% body weight) on the interaction between a powered knee prosthesis and amputee users.

As an important first step toward investigating how mechanically-coupled humans and machines interact in response to changes in the physical environment, here we examined how transfemoral amputees walked and perceived walking effort with 4 load carriage and powered knee mechanics combinations in a 2 by 2 factorial design (Fig. 4). As an inherent property of impedance controllers, both the inter-dependent powered knee impedance parameters and amputee users’ behavior contribute to the overall performance of the powered knee and amputee user. Our results only partially support our hypothesis, as we observed greater reliance on the intact limb and perceived exertion only in the mismatched condition when amputee subjects walked with load. When amputee subjects walked without load, the impedance parameters did not significantly affect amputee subjects’ gait performance and perceived exertion. Total double limb support time only increased with the presence of load. Further, positive intact-limb joint work and center-of-mass velocity did not significantly increase in the mismatched conditions. We will first assess the performance of the powered knee and amputee subjects during the two load-impedance matched conditions together and then each unmatched condition separately.

For the two matched testing conditions in which amputee subjects walked with powered knee impedance parameters tuned for the load they were carrying, prosthetic knee kinematics resembled more normative values. Specifically, during limb extension in late stance, the powered knee maintained a relatively small angle (i.e. close to 0 degrees), which is similar to able-bodied people^32^. Biological leg muscle activity prevents hyper-extension and provides optimal stability during this portion of the gait cycle to prepare for push off^33^.

Interestingly, the addition of load on amputee subjects’ upper body only affected the powered knee prosthesis performance when the weight-bearing prosthetic limb extended in late stance and trailed behind the upper body. During this gait phase, the upper body of the amputee subjects created an external extension moment about the prosthetic knee joint. Carrying the weighted backpack increased these mechanical demands. Without appropriate impedance parameters, the prosthetic knee could not achieve sufficient opposing flexion moment to maintain a stable angle, and the prosthetic knee joint hyper-extended (Fig. 1b, roughly 50% stride). Amputee subjects then relied more on their intact limb (e.g. longer intact-limb stance time), and their overall perceived exertion increased. The intact-side knee and hip absorbed energy (Table 2). To adjust to the hyperextended powered knee, subjects adjusted their inter-limb timing while maintaining a tight coupling of intact limb segments (i.e. kinematics). Specifically, subjects shortened their intact-side step time and lengthened their prosthetic-side step time, resulting in significantly greater step time asymmetry. They may have decreased their intact-side step time to place their intact limb on the treadmill more quickly for stability as they transferred their weight from their prosthetic limb to their intact limb. Subjects placed their intact limb on the treadmill as the prosthetic limb extended in mid-stance, but they did not place their prosthetic limb on the treadmill until the intact limb was fully extended in late stance (Fig. 1). With their intact limb on the treadmill as the prosthetic limb extended in mid-stance (and even earlier in the case of greater step time asymmetry), an increased double support duration may have conveyed more stability as the prosthetic knee unfavorably hyper-extended in late stance and as they subsequently transferred their weight to their intact limb. Double limb support versus single limb support is thought to increase one’s base of support and thus stability^34^. Additionally, because subjects spent more time transferring their weight from the prosthetic limb to their intact limb, they may have also hastened their intact-side step time to “catch up” on the treadmill to maintain a constant walking speed. Matched *load* impedance parameters favorably reduced amputee subjects’ intact-limb stance time, lengthened intact-limb swing time, and increased step time asymmetry. Intact-limb negative work remained the same with load and both parameter sets, likely because energy absorption was required for load carriage, but positive prosthetic-side hip work decreased with matched load parameters.

On the other hand, during the other mismatched condition in which amputee subjects did not carry load but walked with the *load* impedance parameters, the prosthetic knee did not fully extend toward 0 degrees during limb extension in stance phase (Fig. 1a, roughly 50% stride). Though the differences in knee angle and perceived exertion are statistically insignificant, we reason that the slightly greater powered knee angle resulting from mismatched impedance parameters may have contributed to the coinciding minimally greater perceived exertion scores (Fig. 2). Perhaps without the weighted backpack, the amputee subjects’ body weight was not sufficient to overcome the additional flexion moment generated by the prosthetic knee with *load* impedance parameters in order to fully extend the prosthetic knee. Thus, subjects were “pushed” to their intact limb more quickly than preferable, as indicated by their shorter prosthetic-side terminal double support time. Ellis *et al*. (2013) found that able-bodied people incurred greater mechanical and metabolic costs when walking with greater step asymmetry than his/her preferred level of asymmetry^35^, but this similar correlation between subjects’ gait and perceived exertion in our study was only apparent when subjects carried load (Fig. 2, Table 1). We instead observed greater symmetry with this mismatched no load condition. There may be a preferred level of prosthesis torque and asymmetry specific to each amputee user that tuning experts are able to identify during the tuning process. When amputee subjects exhibit improved symmetry beyond this preferred level, they potentially exert more effort to accomplish goals not related to the measures we analyzed here (e.g. reduce socket-residual limb forces, medial-lateral stability).

Removing early stance flexion and extension (i.e. locking the knee during stance) could eliminate the observed adverse effects during stance phase, but stance flexion plays a key role in energy efficiency and shock absorption to protect proximal joints and the lower back^36^. Kaufman *et al*. observed substantial gait performance benefits (e.g. increased reliance on prosthetic limb) with prosthetic knee stance flexion^37^. We consider stance phase knee flexion integral to improving the gait of prosthesis users.

Contrary to what we expected, the amputee subjects did not noticeably adapt their center-of-mass velocity profiles during walking despite large changes in gravitational load (i.e. load carriage, 20% body weight) and powered prosthesis mechanics (i.e. impedance parameters) that were either matched or mismatched to the gravitational load conditions (Fig. 3). Walking with a powered prosthesis that generated normative knee behavior did not result in center-of-mass velocity profiles that were more symmetric or similar to an able-bodied profile. These findings suggest that each amputee subject in our study had a unique and well-learned walking strategy influenced by both the amputee’s characteristics (e.g. height, weight, muscle strength, residual limb length) and functional constraints of their prescribed (i.e. energetically-passive) prostheses that became highly reinforced post amputation^29,38^, (time since amputation ranged from 5–45 years in our study). In this study, cumulative walking time during training with the powered prosthesis at baseline condition (i.e. no load carriage and matched impedance parameters) was approximately 2–3 hours. A previous study looking at above-knee amputees walking with a new prosthesis (i.e. with prosthesis behavior different than their prescribed) found that after 7-8 hours of walking was sufficient for amputees to adapt their kinematics and kinetics to match the mechanical properties of the new prosthesis^39^. Therefore, we suspect that the limited training time with the experimental powered prosthesis in our study was not sufficient for amputees to explore new walking strategies that would noticeably alter their center-of-mass velocity profiles. In future studies where the goal is to determine how amputees adapt to walking with a powered prosthesis, we suggest accelerating the learning process by encouraging exploration and alternate walking strategies.

Each subject walked with the experimental powered prosthesis with differing gait patterns and thus may have adapted to the prosthetic knee and external load changes differently or to differing degrees. For example, when subject 2 carried load, he compensated with both his intact-side hip and ankle joint as evidenced by changes in positive joint work^40^. On the other hand, subject 3 compensated with his intact-side hip, potentially because he wore a shoe lift on his intact foot to walk with our experimental prosthesis with proper joint alignment as determined by a certified prosthetist. This shoe lift may have altered his foot-ankle dynamics^41^, and may be considered in future studies. Many factors such as training time with the powered prosthesis, balance confidence^42^, proprioception, socket fit, muscle strength, body mass/size, posture^43^, and trust in powered devices can have considerable effects on their performance and utilization. Thus, we encourage subject-specific analyses to further investigate potential physical and functional factors of compensatory gait strategies in clinical populations such as transfemoral amputees.

In future studies, we suggest including subjective assessments (e.g. user preference) in addition to Rate of Perceived Exertion. Some subjects felt limited when scoring each trial during testing using only perceived exertion, and may have preferred an additional user preference scale. For example, subject 1 scored both conditions in which she carried load closely based on exertion alone, but she enthusiastically said she felt like she “had an ankle” when she walked with the *load* impedance parameters. Perceived exertion or relieving the intact limb as the dominant limb for support are likely only a subset of amputee’s many goals during gait. This study leaves the open questions of what are amputees’ goals (and relative importance of each) during walking with a powered prosthesis, and how can engineers design prosthesis controllers to optimally achieve these goals.

It is important to understand our results represent the behavior of our small sample size, and may not represent the behavior of the entire amputee population. Rather, our pilot study explores a novel type of human-machine interaction and describes the preliminary need to consider more real-world tasks in prosthetics research. Our results also cannot be interpreted with respect to traditional energetically-passive prostheses, as this comparison was outside of the scope of our study. Powered prostheses behave fundamentally differently, and we believe they are a promising advancement for amputee users. Additionally, our experimental powered knee prosthesis does not have an embedded toque sensor, so we cannot make any claims about the exact impedance applied to the controller. Rather, our testing platform is dependent on amputee-prosthesis performance. Lastly, the tuning process (both in clinics and in our study) is not objective, but rather heuristic and is highly sensitive to the tuning expert’s training and experience. Further exploration and optimization of the tuning process itself may lead to more effective parameters that cannot be discovered manually^8^.

This study highlights the importance of observing prosthesis controller and amputee user behavior in varied environmental conditions representative of users’ daily tasks. The matched prosthetic knee impedance control parameters during load carriage largely improved the robustness of the human-prosthesis system to respond to external load changes and may be useful for daily use. When subjects switched between carrying and not carrying load, the *load* impedance parameters induced smaller gait and perceived exertion changes. However, the tuning expert did not originally arrive at this level of stiffness prior to the subject carrying load, likely because it induced an abnormally flexed prosthetic knee angle, and he did not explore that range. There are a number of impedance parameter settings that can produce the same observable gait performance when walking without load^6^, so adding task demands that a user might typically experience in their daily life (e.g. weighted backpack) during the tuning process may improve user satisfaction. The tuning expert may be able to recognize parameter weaknesses and choose a more robust parameter set for each amputee that perform well across tasks (but perhaps not optimally for every situation). Though, based on our study, tuning experts must not ignore the converse condition (i.e. walking with *load* parameters and removing the backpack) and user feedback.

Alternatively, and perhaps more optimally, transfemoral amputees could use a prosthesis controller than automatically switches to load-specific prosthesis knee mechanics that exploit the utility of powered prostheses (e.g. appropriate flexion torque) for specific environmental demands. However, pre-determining controller settings for every variation of daily tasks would be time-consuming and tedious. With the advancement of adaptive controllers, these load-specific mechanics may not have to be manually prescribed, but rather learned by the controller. For example, with the use of reinforcement learning, powered knee prostheses can automatically adjust the impedance parameters to generate a preferred knee angle^44^. This type of controller may be able to change the impedance parameters to avoid hyperextension when the amputee user carries load (as observed in this study). Future research in both in-clinic tuning procedure improvements and environment-adaptive prosthetic knee controllers (e.g. reliability, accuracy) may enable transfemoral amputees to ambulate with improved performance and satisfaction in their daily life.

## Conclusion

In this study, we evaluated how transfemoral amputees and a powered knee with pre-determined mechanics interact under the influence of gravitational load. Load-related adverse effects such as increased reliance on the intact limb and perceived exertion increased when subjects carried load and decreased with the appropriate powered knee mechanics pre-determined for load carriage. Based on our results and amputee subjects’ subjective feedback during this study, we believe tuning experts/clinicians can prescribe more robust and preferable prosthesis control parameters if the amputee users are exposed to more tasks that alter the dynamics of the human-prosthesis system (e.g. load carriage) in the clinic during the typical tuning procedure. Perhaps in the long run, researchers can develop robust yet highly adaptive controllers that automatically adjust the prosthesis mechanics according to both the physical environmental demand and the individual user’s engagement with the prosthesis.

## Methods

### Subjects

Five transfemoral amputees of functional level K3 (characteristic of typical community ambulators (Table 3) participated in our study. All subjects provided written, informed consent to participate in our study approved by the The Institutional Review Board of the University of North Carolina at Chapel Hill, and we conducted our study in accordance with the relevant guidelines and regulations. We recruited subjects that have been using their prosthesis for more than one year and typically carry objects such as school or work bags. Both male and females walk with similar gait characteristics during load carriage^19^, so we included both genders. We excluded people with comorbities that may affect ambulation with our experimental device. A certified prosthetist aligned our powered prosthesis for each amputee subject, and we maintained the same alignment for every visit. We added height (i.e. extra components) at the prosthetic joints or the intact limb (i.e. shoe lift) to maintain the subjects’ pelvis and knee joint centers as level as possible.

**Table 3:** Subject characteristics. Body weight includes the subject’s prescribed prosthesis.

Subject	Gender	Body weight	Height	Age	Since amputation	Amputated side	Prescribed prosthesis	Shoe lift for intact foot
1	Female	56 kg	1.70 m	27 years	Congenital (27 years)	Right	Össur Total Knee	6 cm
2	Male	66 kg	1.83 m	20 years	5 years	Right	Ottobock Genium	0 cm
3	Male	66 kg	1.65 m	61 years	13 years	Left	Ottobock C-Leg	8 cm
4	Male	91 kg	1.80 m	57 years	45 years	Left	Ottobock C-Leg	0 cm
5	Male	95 kg	1.88 m	29 years	Congenital (29 years)	Left	Freedom Innovations Plié 2	4 cm

### Experimental setup

All subjects walked at 0.6 m/s with our experimental powered knee prosthesis^45^ and carbon high-performance foot (1E56 Axtion, Otto Bock, Germany) with and without a weighted backpack containing 20% of their body weight. Our selected backpack weight was low compared to military-grade weight used in other load-carriage studies, but it is more representative of daily living and appropriate for our K3 (versus K4) subjects, and other studies have used similar weights^19,21,26,46,47^. We recorded ground reaction forces from a split-belt treadmill (1000 Hz, Bertec Corp., Columbus, OH, USA) and full-body kinematics using an 8-camera motion capture system (42 markers, 100 Hz, VICON, Oxford, UK).

### Prosthetic knee impedance control

Our experimental powered knee prosthesis used a finite state machine with five predefined gait phases: stance flexion, stance extension, pre-swing, swing flexion, and swing extension. The motor generated a different amount of torque, *t*_*p*_, during each gait phase according to the predetermined impedance control parameters (i.e. stiffness, *k*, equilibrium position, *θ*_*e*_, damping, *b*), real-time measurements from the powered knee prosthesis (i.e. angle, *θ*_*p*_, angular velocity, ${\mathop{\theta }\limits^{.}}_{p}$, ground reaction force), and the fundamental impedance control equation (1). We measured the prosthetic knee angle using an angle sensor embedded at the prosthetic knee joint, computed the derivative for the angular velocity, and collected the ground reaction force from a load cell at the distal end of the pylon.

1 $${t}_{p}=k\cdot ({\theta }_{p}\,-\,{\theta }_{e})+b\cdot {\mathop{\theta }\limits^{.}}_{p}$$

We adjusted the subject-specific ground reaction force thresholds that affect phase transitions (particularly swing extension to stance flexion)^45^ according to the total mass of the subject and backpack, so the prosthesis phase transition timings remained consistent.

### Experimental design

Prior to testing, a tuning expert (well-versed in gait analysis and our experimental prosthesis controller) determined favorable prosthesis impedance control parameter sets for each subject as he/she walked with and without load. To determine these tuned parameter sets, the subject initially walked with a standard (i.e. not subject- or load-specific) set of impedance parameters to become familiar with the testing environment. The expert then fine-tuned the control parameters based on his visual observations of the subject’s gait performance and verbal feedback. The subject rested between adjustments to prevent fatigue. We based this tuning process on the traditional tuning process in a clinic^4,48^. Each subject practiced walking (without carrying load) with the experimental prosthesis set to his/her tuned control parameters for no load over the course of four or more 2-hour visits to our laboratory (cumulative walking time estimated at 2–3 hours) and until he/she felt comfortable walking at 0.6 m/s (i.e. average preferred speed with our device, and slightly slower than transfemoral amputees’ preferred walking speed of 0.9 m/s^49^) without using the treadmill handrails or body-weight support for assistance. During a 2- to 3-hour visit the day before testing, we reset the impedance parameters to the standard setting, and amputee subjects practiced walking with and without load (randomized order between subjects) while the tuning expert determined the tuned control parameters for no load and load. Each subject’s tuned *no load* control parameters were similar to the parameters he/she practiced walking with, but re-tuning accounted for any adaptation or physiological changes from training.

During testing, the subject walked with and without the weighted backpack and both tuned prosthesis impedance parameter sets (Fig. 4). We repeated these 4 conditions 3 times, for a total of 12 2-minute trials. We treated the 3 replicate trials as independent measures, randomized the sequence of the 12 total trials, and blinded the subject to the prosthesis settings to preclude confounding effects of fatigue, training time, and subject bias. After each trial, we administered a 3-minute (or more) break and recorded the subject’s Rate of Perceived Exertion (RPE) with the modified Borg CR10 scale^50^ that has previously been used for load carriage^21^. Perceived exertion indicates relative subjective preference by capturing psychological and physiological responses related to effort.

### Data processing

We analyzed 10 consecutive strides from the last 30 seconds of each trial, and these strides were selected between scuffs and treadmill handrail touches. We used standard analysis software (Visual 3D, C-Motion, Inc., Germantown, MD, USA) to estimate sagittal-plane joint kinematics and kinetics and two-dimensional center-of-mass trajectories (i.e. anterior-posterior and superior-inferior position and velocity). We calculated positive and negative joint work by integrating the positive and negative portions of the joint power trajectories. To appropriately estimate joint kinetics and the center of mass trajectories, we manually adjusted the inertial properties of the prosthesis segments and created an extra segment for the weighted backpack. We filtered the motion and force-plate data using a fourth-order Butterworth low-pass filter with a cutoff frequency of 7.5 Hz. We baseline-corrected the force-plate signals and identified gait events using a force threshold of 20 N. We manually calculated all temporal parameters (i.e. stride time, stance time, swing time, initial double support time, terminal double support time, and step time) from the gait events. We defined step time as contralateral heel strike to ipsilateral heel strike. We normalized all data to the stride time of the corresponding limb and kinetic data to the subjects’ total mass including the weighted backpack. To evaluate step time and center-of-mass forward velocity asymmetry, *s*, we used a standard asymmetry index^51^ (2). The intact limb measures are denoted by *i*, prosthetic limb measures are denoted by *p*, and step time or center-of-mass forward velocity for the corresponding limb is denoted by *x*.

2 $$s=({x}_{p}-{x}_{i})/(({x}_{p}+{x}_{i})\cdot {\rm{0.5}})\cdot 100$$

### Statistical tests

We averaged the three repeated trials for each condition within subjects and used two-way repeated-measures ANOVA to test for main effects of and interactions between independent factors (i.e. load and prosthesis impedance control parameters), using an alpha level of 0.05. We treated subject as a random blocking factor and removed 2 outliers (Supplementary Methods). When we found a significant effect, we tested for statistical differences within load and impedance parameter conditions using Tukey’s honestly significant difference test (*α* = 0.05). In the presence of significant interaction effects, we ignored significant main effects. We included the outliers in figures for representation of the full data set.

## Electronic supplementary material

Supplementary Information
