# Supplementary material for: Interactions Between Transfemoral Amputees and a Powered Knee Prosthesis During Load Carriage
Source: Sci Rep. 2017 Nov 3;7:14480. doi: 10.1038/s41598-017-14834-7 (PMC5670174; doi:10.1038/s41598-017-14834-7)
Supplement: Supplementary file 1 — Supplementary Information [file 41598_2017_14834_MOESM1_ESM.pdf]

Supplementary Information

Interactions Between Transfemoral Amputees and a Powered Knee Prosthesis During Load Carriage

Andrea Brandt, Yue Wen, Ming Liu, Jonathan Stallings, and He (Helen) Huang

University of North Carolina and North Carolina State University, Joint Department of Biomedical Engineering, Chapel Hill and Raleigh, 27514 and 27695, USA

Table S1. The tuning expert prescribed greater knee stiffness and/or equilibrium position impedance parameters for all users when they carried load. Stiffness and equilibrium position parameter changes for each subject are listed for the stance extension phase only. Subjects with asterisks (*) received tuning in other phases such as swing extension, but the changes were minor compared to the stance extension phase. Relative change is the change of each parameter relative to other subjects (i.e. normalized to the greatest change of that parameter across subjects).

Subject ID	Stiffness – No load	Stiffness – Load	Relative Change	Equilibrium Position – No load	Equilibrium Position – Load	Relative Change
1*	3.3	3.3	**0%**	6	10	**100%**
2*	2.7	3.3	**100%**	10	12	**50%**
3*	2.3	2.7	**67%**	14	14	**0%**
4	3.8	3.8	**0%**	12	16	**100%**
5	3.1	3.2	**17%**	11	14	**75%**

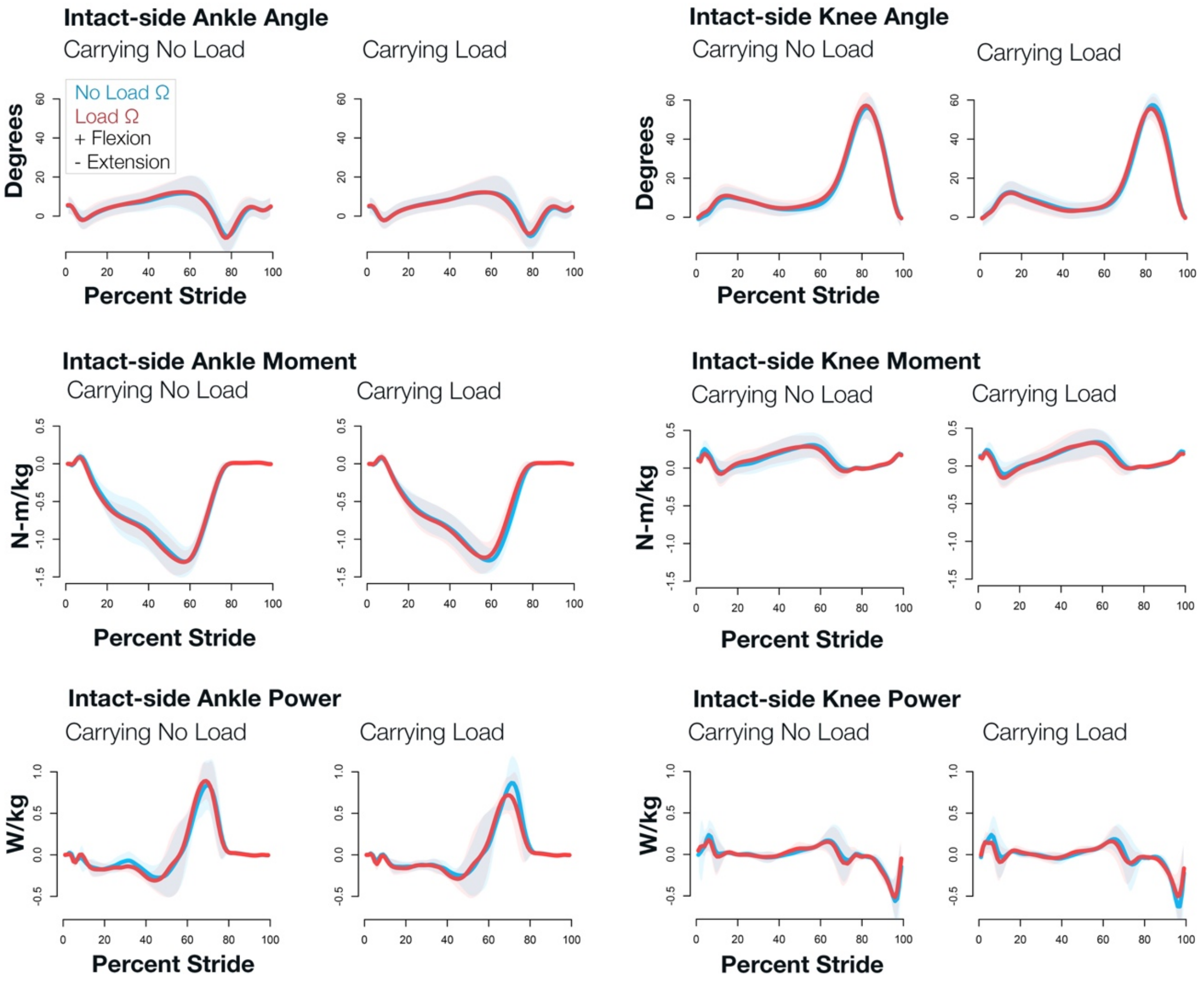

Figure S1. Intact joint kinematics and kinetics. All kinetics were normalized to total mass (body mass and backpack mass). Blue lines indicate conditions in which amputee subjects walked with *no load* impedance parameters, and red lines indicate conditions in which they walked with *load* impedance parameters. For angles and moments, positive values indicate flexion, and negative values indicate extension. For power, positive values indicate power generation, and negative values indicate power absorption.

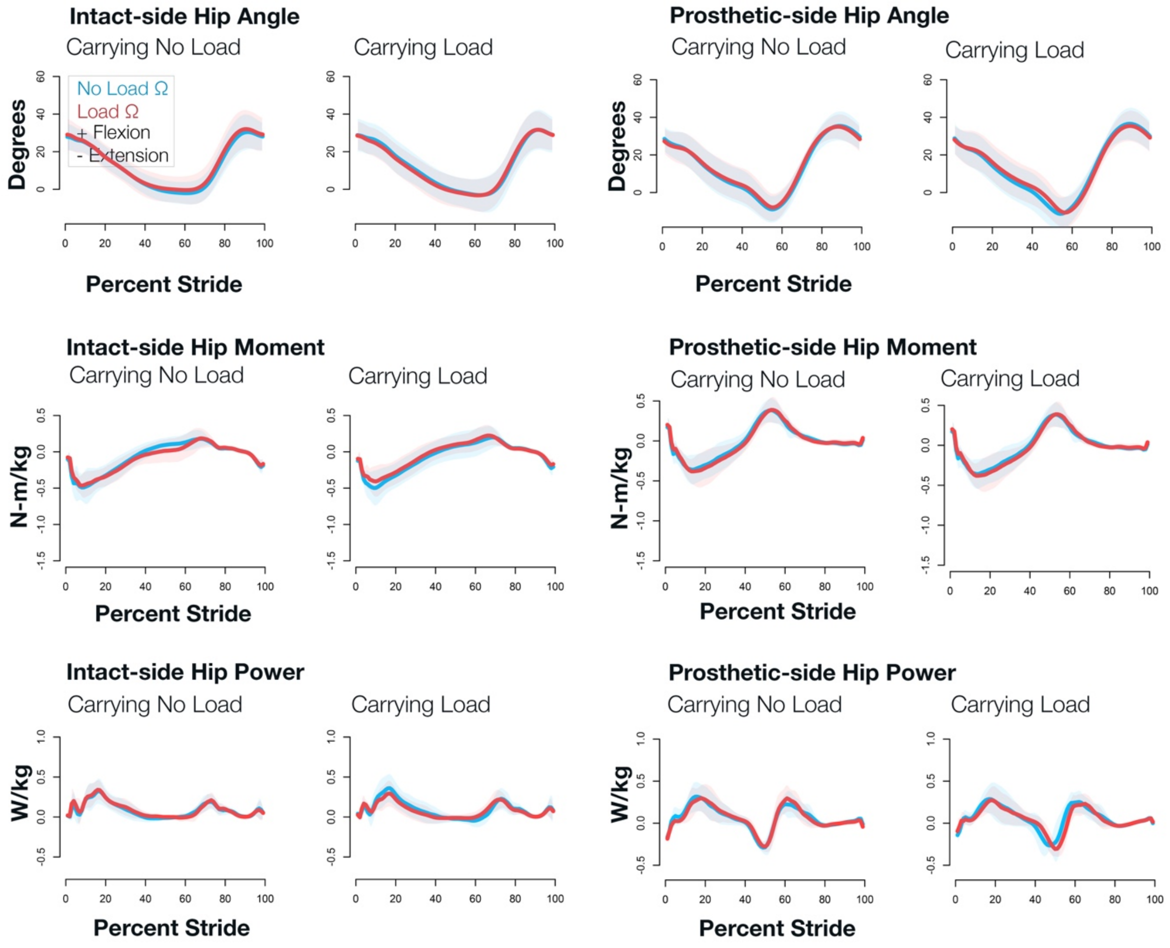

Figure S2. Intact joint kinematics and kinetics. All kinetics were normalized to total mass (body mass and backpack mass). Blue lines indicate conditions in which amputee subjects walked with *no load* impedance parameters, and red lines indicate conditions in which they walked with *load* impedance parameters. For angles and moments, positive values indicate flexion, and negative values indicate extension. For power, positive values indicate power generation, and negative values indicate power absorption.

Methods

We methodically reviewed the distribution and normal probability of the conditional residuals after performing two-way repeated measures ANOVA with subject as a random factor. Four measures (i.e. perceived exertion, positive intact ankle work, positive intact-side hip work, and negative prosthetic-side hip work) failed the normality test (Shapiro-Wilk, $p<0.01$) due to a noticeable outlier. All 4 outliers were different trials, and only appeared as an outlier in one specific measure.

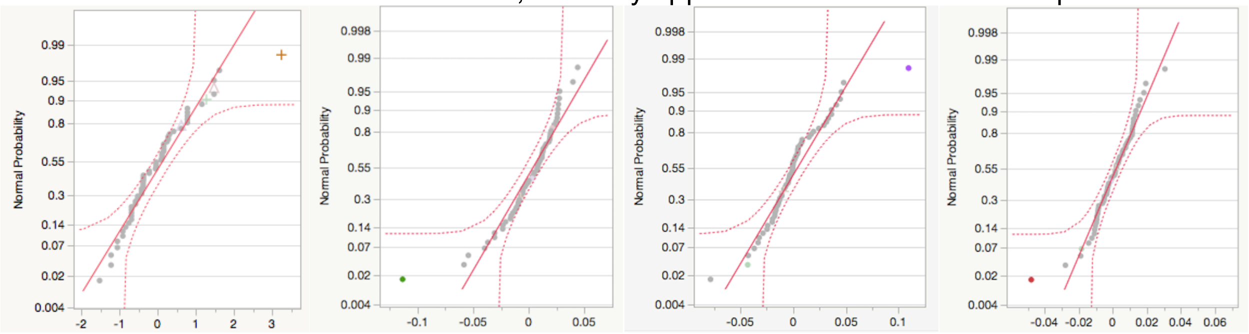

Figure S3. Conditional residuals versus normal probability with 4 outliers identified. Perceived exertion ($p<0.001$), positive intact ankle work ($p<0.001$), positive intact-side hip work ($p=0.002$), and negative prosthetic-side hip work ($p=0.016$) are plotted in order with each outlier colored.

We excluded the first two outlier trials identified and included the remaining 2 trials. The ANOVA results did not significantly change with the inclusion/exclusion of the last 2 outliers (Table 2), and the complete exclusion of these trials may remove valuable information because these trials were not outliers for any other measure. The exclusion of the first 2 outliers allowed both perceived exertion and positive intact ankle work to have an approximately normal distribution (below).

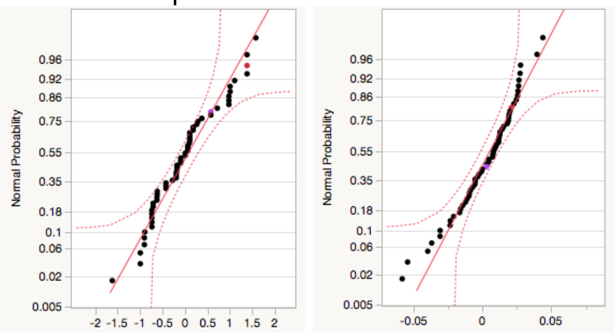

Figure S4. Conditional residuals versus normal probability with 2 outliers removed. Perceived exertion ($p=0.048$), positive intact ankle work ($p=0.074$) are plotted in order.

Table S2. ANOVA results with individual outlier inclusion/exclusion. Bold font indicates significance at 0.05 level.

	Load main effect	Impedance main effect	Interaction effect
Perceived exertion (included)	0.159	**<0.001**	**0.006**
(excluded)	**0.046**	**<0.001**	**0.005**
Positive ankle work (included)	0.621	0.158	0.065
(excluded)	0.198	**0.015**	0.119
Positive intact-side hip work (included)	0.481	0.538	0.070
(excluded)	0.387	0.721	0.105
Negative prosthetic-side hip work (included)	0.378	**0.016**	0.740
(excluded)	0.520	**0.011**	0.921
